# In Vitro Evaluation of Poly(lactide-co-glycolide) In Situ Forming Gels for Bedaquiline Fumarate Salt and Pharmacokinetics Following Subcutaneous Injection in Rats

**DOI:** 10.3390/pharmaceutics13081231

**Published:** 2021-08-10

**Authors:** Sandy Van Hemelryck, Rani Wens, Hannelore van Poppel, Milou Luijks, Koosha Shahidi, Marcin Marczak, Ariane Kahnt, René Holm, Erik Mannaert, Peter Langguth

**Affiliations:** 1 Clinical Pharmacology and Pharmacometrics, Janssen Research and Development, Johnson & Johnson, Turnhoutseweg 30, 2340 Beerse, Belgium; raniwens@hotmail.com (R.W.); hannelorevp@hotmail.com (H.v.P.); milouluijks@gmail.com (M.L.); kshahidi@its.jnj.com (K.S.); emannaer@its.jnj.com (E.M.); 2 Faculty of Pharmaceutical Sciences, University of Leuven, Herestraat 49, 3000 Leuven, Belgium; 3 Department of Biomedical Sciences, Faculty of Pharmaceutical, Biomedical and Veterinary Sciences, University of Antwerp, Universiteitsplein 1, 2610 Wilrijk, Belgium; 4 Analytical Development, Janssen Research and Development, Johnson & Johnson, Turnhoutseweg 30, 2340 Beerse, Belgium; mmarczak@its.jnj.com; 5 Bioanalysis, Janssen Research and Development, Johnson & Johnson, Turnhoutseweg 30, 2340 Beerse, Belgium; kariane@its.jnj.com; 6 Drug Product Development, Janssen Research and Development, Johnson & Johnson, Turnhoutseweg 30, 2340 Beerse, Belgium; rholm@its.jnj.com; 7 Department of Physics, Chemistry and Pharmacy, University of Southern Denmark, Campusvej 55, DK-5230 Odense, Denmark; 8 Pharmaceutical Technology and Biopharmaceutics, Johannes Gutenberg University, Staudingerweg 5, 55128 Mainz, Germany; langguth@uni-mainz.de

**Keywords:** in situ forming gels, injectable, bedaquiline, in vitro release, pharmacokinetics, sustained release, porosity, diffusion, dissolution, polymer erosion

## Abstract

This study evaluated in vitro and in vivo drug release of bedaquiline from in situ forming gels (ISGs) containing 200 mg eq./g bedaquiline fumarate salt prepared with four different grades of poly(d,l-lactide) (PDLLA) or poly(d,l-lactide-co-glycolide) (PLGA) with a lactide/glycolide ratio of 50/50 or 75/25 and acid (A) or ester (E) end-capping in *N*-methyl-2-pyrrolidone at a polymer/solvent ratio of 20/80% (*w*/*w*). Mean in vitro drug release in 0.05 M phosphate buffer pH 7.4 with 1% (*w*/*v*) sodium lauryl sulphate was 37.3, 47.1, 53.3, and 62.3% within 28 days for ISGs containing PLGA5050A, PDLLA, PLGA7525A, and PLGA7525E, respectively. The data suggested that drug release was primarily controlled by precipitated drug redissolving, rather than polymer erosion. In vivo pharmacokinetic profiles after subcutaneous injections in rats were comparable for all ISGs (mean half-lives (t_1/2_) ranged from 1411 to 1695 h) and indicated a sustained drug release when compared to a solution of bedaquiline fumarate salt in polyethylene glycol 400/water 50/50% (*v*/*v*) (mean t_1/2_ of 895 h). In conclusion, PLGA or PDLLA-based ISGs have shown potential for parenteral sustained delivery of bedaquiline, suggesting further preclinical and clinical studies. From a formulation point of view, this case example highlights the importance of the interplay between drug solubility in biological media and dissolution of drug precipitates, which, in addition to the incorporation of diffusion controlling polymers, governs the release of the active drug.

## 1. Introduction

Tuberculosis (TB), caused by the bacillus *Mycobacterium tuberculosis*, is one of the deadliest infectious diseases worldwide. In 2019, an estimated 10.0 million new TB cases were reported with approximately 1.4 million deaths [1]. Bedaquiline, an inhibitor of the mycobacterial adenosine triphosphate (ATP) synthase proton pump involved in the energy production of mycobacteria, is widely used in the treatment of multidrug resistant TB (MDR-TB) [2,3]. The compound has been marketed as oral tablets under the brand name Sirturo^®^ since 2012 [4]. The long treatment duration (6 to 20 months) with frequent drug administrations may lead to early discontinuation or poor adherence, resulting in reduced cure rates and emerging resistance. Therefore, a long-acting injectable (LAI) formulation, providing sustained therapeutic exposures of bedaquiline with reduced frequency in dosing, can help to improve treatment outcomes [5].

Recent studies have demonstrated favourable pharmacokinetic profiles and antiviral activity after intramuscular or subcutaneous injection of bedaquiline LAI microsuspensions in rodents [6,7]. The high lipophilicity (log P 7.3), low aqueous solubility (<0.005 mg/mL at pH 7.4), and high potency against M. tuberculosis (minimal inhibitory concentration of 0.03 µg/mL) make bedaquiline a suitable candidate for this type of LAI formulation [6,7]. However, LAI microsuspensions have some disadvantages: for example, their complex and costly manufacturing process and physical stability challenges related to particle size growth. An interesting alternative for TB treatment, especially for developing countries where low cost of goods is an important factor, could be LAI in situ forming gels (ISGs) containing bedaquiline. In these systems, the drug substance is suspended or solubilized in an organic or aqueous biocompatible polymeric solution. When the solution or suspension is injected in vivo, the polymer forms a gel in response to physiological stimuli (e.g., temperature, pH), chemical reactions (e.g., polymerization), solvent exchange, or swelling at the injection site [8]. ISGs prepared as a solution, do not require high pressure or shear to reduce particle size of the compound as in top-down processes applied for LAI microsuspensions. Instead, the drug is dissolved in the polymer solution allowing for final sterilization by filtration and filling into vials or pre-filled syringes, resulting in a very simplified process with a potential for a lower contamination risk during manufacturing and upon preparation before injection. It should be noted, though, that chemical stability issues may require that the drug and polymer solutions be filled separately and mixed right before use.

Several commercial ISGs containing poly(lactide-co-glycolide) (PLGA) as a polymer have been approved for human use (e.g., Eligard^®^, Atridox^®^, and Sublocade^®^), providing sustained drug release ranging from 1 week to 6 months, making them an interesting LAI system to consider for bedaquiline [9,10,11]. PLGA ISGs belong to the phase inverting systems that form a gel upon solvent exchange [8]. Contact with an aqueous environment or body fluids triggers a phase inversion process in the polymer solution resulting in polymer precipitation [12]. The release mechanism of a drug from phase inverting systems is a result of matrix solidification, drug diffusion through pores and/or polymer matrix, and polymer erosion [12,13,14]. Factors such as drug properties, polymer grade, solvent type, and concentration of each of the components have been shown to influence these processes and should be carefully selected in order to obtain the desired release profile [12,15]. PLGA polymers exist in different grades: their lactide/glycolide (L/G) ratio, molecular weight (or inherent viscosity), and end capping determine their hydrophobicity and degradation rate [14,15]. In general, higher lipophilicity and higher viscosity lead to slower water ingress and slower hydrolysis [16,17]. As PLGA polymers are insoluble in water, they are solubilized in organic solvents to prepare ISGs. If water-miscible organic solvents (e.g., *N*-methyl-2-pyrrolidone (NMP)) are used, ISGs undergo a fast phase inversion when injected in vivo, typically resulting in a high drug burst release and high gel porosity. Water-immiscible solvents (e.g., triacetin) lead to a prolonged solidification process with reduced burst, lower porosity, and decreased diffusivity.

The literature on ISGs evaluating the effect of formulation variables on both in vitro and in vivo drug release is sparse, and most published case examples focus on ISGs containing different polymer concentrations, solvents, and/or additives [18,19,20,21,22,23,24,25,26,27,28]. Only limited information is available for ISGs prepared with variations in polymer grade (i.e., L/G ratio, end capping, molecular weight), and confirmation of the observed differences in in vitro drug release by in vivo studies is often lacking [21,23,27,28,29,30,31,32,33]. The purpose of the present study was to develop PLGA-based LAI ISG concept formulations for bedaquiline and to evaluate the impact of polymer properties on in vitro and in vivo drug release. Four polymer grades with different L/G ratios, resulting in different erosion rates, were selected to produce a variety of drug release rates. Additionally, the ISG concept formulations were designed to provide high drug concentrations and doses leading to long-term therapeutic exposures after subcutaneous (SC) injection. Drug solubility in the formulations was therefore maximized by selecting the bedaquiline fumarate salt over the free base and by minimizing the polymer/solvent ratio to 20/80% (*w*/*w*), based upon suggestions from the literature [18,19,20,21].

## 2. Materials and Methods

### 2.1. Materials

Bedaquiline fumarate salt and 6-deuterium-labelled bedaquiline were manufactured internally at Janssen. PLGA5050A (L/G ratio 50/50, acid terminated, inherent viscosity (IV) 0.48 dL/g), PLGA7525A (L/G ratio 75/25, acid terminated, IV 0.48 dL/g), PLGA7525E (L/G ratio 75/25, ester terminated, IV 0.35 dL/g), PDLLA (poly(d,l-lactide), acid terminated, IV 0.71 dL/g), lactide, and glycolide were sourced from Ashland (Covington, KY, USA). Polyethylene glycol 400 (PEG400) was purchased from Clariant (Frankfurt am Main, Germany). NMP was sourced from Acros Organics (Geel, Belgium) and water for injections from Sterop Laboratoria (Brussels, Belgium) or Baxter (Lessines, Belgium). All other chemicals were purchased from commercial sources as reagent grade.

### 2.2. Formulation Preparations

Table 1 provides an overview of the formulations prepared for the in vitro and/or in vivo assessments described in this study.

ISG formulations 1, 2, 3, and 4 containing 242 mg/g bedaquiline fumarate salt (equivalent to 200 mg/g bedaquiline free base, and further referred to as 200 mg eq./g bedaquiline fumarate salt) were prepared in PLGA5050A/NMP, PLGA7525A/NMP, PLGA7525E/NMP, and PDLLA/NMP 20/80% (*w*/*w*), respectively. First, the polymer was dissolved in NMP, and then bedaquiline fumarate salt was added and stirred until a clear solution was obtained.

PEG400 solution formulation 5 containing 6.05 mg/mL bedaquiline fumarate salt (equivalent to 5 mg/mL bedaquiline free base, and further referred to as 5 mg eq./mL bedaquiline fumarate salt) was prepared in PEG400/water 50/50% (*v*/*v*), by dissolving the drug substance in PEG400, followed by dilution with water.

Formulations administered to rats were end-sterilized by filtration through a 0.2 µm Whatman filter (ISGs) or 0.22 µm Sterivex (PEG400 solution formulation) sterile filter.

### 2.3. In Vitro Evaluations

#### 2.3.1. In Vitro Release Study

The in vitro release study for ISG formulations 1 to 4 was performed in duplicate. An 8-vessel Distek USP paddle apparatus 2 was used, containing a dissolution medium of 900 mL 0.05 M phosphate buffer pH 7.4 with 1% (*w*/*v*) sodium lauryl sulphate (SLS) per vessel, maintained at 37 °C. A total of 0.5 g of the formulations to be tested was added to cylindric plastic cups, and 1 mL of buffer was transferred from the dissolution vessels to the cups. The formulations were allowed 10 min to form a gel in the cups, and those cups, containing 0.5 g formulation and 1 mL of buffer, were then transferred to the dissolution vessels in a staggered way (30 s in between each cup). After all cups were added to the dissolution vessels, the paddle was set to stir at 50 rpm. A total of 3.5 mL of dissolution medium was sampled with a 5 mL syringe through a Distek needle at 0.25, 0.5, 1, 2, 4, 8, 24, 48, 144, 216, 312, 384, 480, 552, and 672 h after addition of the formulations to the dissolution vessels. A 30 mm × 0.2 µm Spartan Whatman filter was used to filter the collected dissolution medium, and samples were stored at room temperature until analytical testing (see Section 2.3.2). Mean in vitro release profiles were compared by calculation of similarity factors (f2) up to 48 and 672 h to evaluate differences in burst and overall release, respectively [34].

#### 2.3.2. Analytical Method for the In Vitro Release Study

For the in-house developed analytical method, a Waters Acquity H-Class UHPLC system (Zellik, Belgium) with ultraviolet (UV) absorbance detector and Waters Empower 3 software was used to quantify bedaquiline in the in vitro release samples. The samples were injected (injection volumes ranged from 1 to 25 µL) onto a Waters Acquity CSH C18 1.7 µm 2.1 × 50 mm column maintained at 45 °C. The detection wavelength was set at 334 nm. Mobile phases (A) 0.1% (*v*/*v*) trifluoroacetic acid (TFA) in water and (B) 0.05% (*v*/*v*) TFA in acetonitrile (ACN) were prepared and eluted via the following gradient, mixing (A) and (B), at a flow rate of 0.6 mL/min: the percentage of (B) was increased from 10% to 90% during the first minute, kept stable at 90% for 0.5 min, was reduced again to 10% in 0.5 min, and then kept stable at 10% for another minute, resulting in a total run time of 3 min.

#### 2.3.3. Scanning Electron Microscopy and Energy-Dispersive X-ray Spectroscopy

Scanning electron microscopy (SEM) and energy-dispersive X-ray spectroscopy (EDX) analyses were performed for ISG formulations 1 and 2 to evaluate the effect of L/G ratio on morphology and elemental distribution. Sample preparation was based on a method described by Ahmed et al. [18]. For both formulations, spherical ISGs were prepared by releasing droplets of formulation through a funnel into 0.05 M phosphate buffer with 1% (*w*/*v*) SLS (pH 7.4). After storage for 6 days at room temperature, the spherical gels were removed from the buffer, frozen on dry ice in vials for about 15 min, and vacuum dried overnight. SEM images of the surface and cross-sections of the dried gels, sputter coated with gold/palladium (Au/Pd), were taken with a Zeiss Sigma 300 VP field emission scanning electron microscope, operated at 5 kV, using a secondary electron detector (SE2). Elemental analysis was performed with a Bruker XFlash 6|60 EDX detector at 20 kV.

### 2.4. In Vivo Pharmacokinetic Study in Rats

#### 2.4.1. Animals

Sprague Dawley male rats were supplied by Charles River (Sulzfeld, Germany). At the start of the pharmacokinetic study, the rats weighed 350 to 450 g and were 9 to 11 weeks old. Polysulfone cages with corn cob bedding material were used for group housing in airconditioned (20–24 °C) rooms with a 12 h light cycle. To enrich the environment, rats had access to Aspen wood block (Datesand, UK) and Rodent retreat (Bio-Serv, Flemington, NJ, USA). An acclimatization period of at least 4 days was applied before starting the pharmacokinetic study. Food and water were available ad libitum throughout the study.

The guidelines of the Janssen Pharmaceutica (Beerse, Belgium) Animal Ethics Committee, the local Belgium laws controlling the use of experimental animals, and the EC Directive 2010/63/EU were followed.

#### 2.4.2. Pharmacokinetics

Based on body weight, 15 rats were divided into 5 groups (3 animals per group) (Table 2). Rats of groups 1 to 4 received a dorsal SC injection of 0.4 mL/kg or a dose of 96.8 mg/kg bedaquiline fumarate salt (equivalent to 80 mg/kg bedaquiline free base, and further referred to as 80 mg eq./kg bedaquiline fumarate salt) of ISG formulations 1 to 4, respectively. In group 5, rats received a dorsal SC injection of 0.8 mL/kg of formulation 5, corresponding to a dose of 4.84 mg/kg bedaquiline fumarate salt (equivalent to 4 mg/kg bedaquiline free base, and further referred to as 4 mg eq./kg bedaquiline fumarate salt).

At time points ranging from 0.5 h to 168 days after dosing, 32 µL of blood was collected via the tail vein into Vitrex micro hematocrit tubes “soda lime glass” containing potassium ethylenediaminetetraacetic acid (K2.EDTA). Samples were placed on melting ice until centrifugation for approximately 10 min at 1500× *g* and 5 °C. Plasma samples of 10 µL were collected with Vitrex end to end pipettes in FluidX tubes and were stored at −20 °C until bioanalysis (see Section 2.4.3). Pharmacokinetic parameters were calculated by non-compartmental analysis using Phoenix™ WinNonlin^®^ (Certara, Princeton, NJ, USA). A two-tailed homoscedastic t-test was applied for statistical comparisons between groups.

#### 2.4.3. Bioanalytical Method for Pharmacokinetics

The bioanalytical method was developed internally at Janssen. Plasma samples collected during the in vivo study in rats (see Section 2.4.2) were processed by washing out the Vitrex end to end pipettes using 20 µL methanol and 400 µL internal standard solution (5 ng/mL of 6-deuterium labelled bedaquiline in acetonitrile/water 80/20% (*v*/*v*)) into the FluidX tubes. Bedaquiline calibration standards of 0.4 to 1000 ng/mL were prepared in rat plasma and processed in the same way as the study samples. The FluidX tubes were shaken for 10 min using an orbital shaker and were centrifuged for 3 min at 2500× *g*. The supernatant (150 µL) was transferred to a 96-deepwell plate to determine the levels of bedaquiline after injection in a liquid chromatography-tandem mass spectrometry (LC-MS/MS) system. A Shimadzu LC30AD HPLC instrument with an SIL-HTC autosampler (Shimadzu Scientific Instruments, Columbia, MD, USA) and a Waters BEH C18 50 × 2.1 mm, 1.7 µm column maintained at 50 °C was coupled to an API4000™ or 5500™ triple quadrupole mass spectrometer (AB Sciex, Toronto, ON, Canada) equipped with Turbo Ionspray source operated at 400 °C. Mobile phases (A) 0.01 M ammonium formate pH 4.0 and (B) methanol were eluted via the following gradient, mixing (A) and (B), at a flow rate of 0.6 mL/min: the percentage of (B) was increased from 55% to 80% during the first 3 min, was further increased to 98% in 0.01 min, and kept stable at 98% for 0.99 min. Thereafter, the percentage of (B) was again reduced to 55% in 0.01 min and kept stable at 55% for 0.99 min, resulting in a total run time of 5 min. The MS was operated in the positive ion mode using the TurboIonSpray™-interface (electrospray ionization) and was optimized for the quantification of bedaquiline. Multiple reaction monitoring (MRM) was applied with transitions of m/z 555.2 → 58 for bedaquiline and m/z 561.2 → 64 for the 6-deuterium labelled internal standard and a collision energy of 71 eV. The LC-MS/MS results of the calibration standards were used to generate a calibration curve: peak area ratios of bedaquiline to its internal standard were plotted versus corresponding bedaquiline concentrations, and a linear regression model with 1/x^2^ weighting was fitted to these data. Bedaquiline concentrations of the study samples were calculated by interpolation from the calibration curve.

#### 2.4.4. Clinical Observations

Illness, abnormal behaviour or unusual appearance, untoward clinical signs, toxic or pharmacological response, and moribund state or mortality were checked daily for each rat in groups 1 to 5.

## 3. Results

### 3.1. In Vitro Evaluations

#### 3.1.1. In Vitro Release Study for 200 mg eq./g Bedaquiline Fumarate Salt in Polymer/NMP 20/80% (w/w)

**Figure 1:**
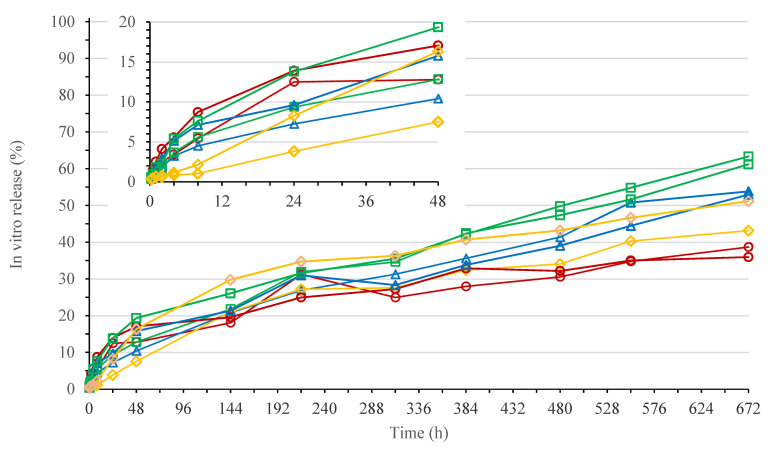
In vitro release of bedaquiline for in situ forming gel (ISG) formulations 1 to 4 (individual profiles for *n* = 2). Red circle: ISG formulation 1 (200 mg eq./g bedaquiline fumarate salt in PLGA5050A/NMP 20/80% (*w*/*w*)); blue triangle: ISG formulation 2 (200 mg eq./g bedaquiline fumarate salt in PLGA7525A/NMP 20/80% (*w*/*w*)); green square: ISG formulation 3 (200 mg eq./g bedaquiline fumarate salt in PLGA7525E/NMP 20/80% (*w*/*w*)); orange diamond: ISG formulation 4 (200 mg eq./g bedaquiline fumarate salt in PDLLA/NMP 20/80% (*w*/*w*)).

The in vitro release profiles of ISG formulations 1 to 4 are shown in Figure 1. The formulations were prepared with the same bedaquiline fumarate salt concentration of 200 mg eq./g and polymer/NMP ratio of 20/80% (*w*/*w*), using four different polymer grades.

Burst release (within 48 h) was quite variable within formulations, with the highest value (mean for *n* = 2) observed for formulation 3 (16.1%), followed by formulation 1 (14.9%), 2 (13.1%), and 4 (11.9%), respectively. Similarity factors (f2) calculated for timepoints up to 48 h ranged from 71 to 93, indicating no significant differences in burst across formulations. After 672 h (28 days), the percentage of drug released in the dissolution medium (mean for *n* = 2) decreased from formulation 3 (62.3%) to formulation 2 (53.4%), 4 (47.1%), and 1 (37.3%), respectively. Similarity factors (f2) calculated for the full in vitro release profiles ranged from 45 to 72 and indicated a difference for formulation 1 versus 3 only (f2 < 50).

#### 3.1.2. Scanning Electron Microscopy and Energy-Dispersive X-ray Spectroscopy

**Figure 2:**
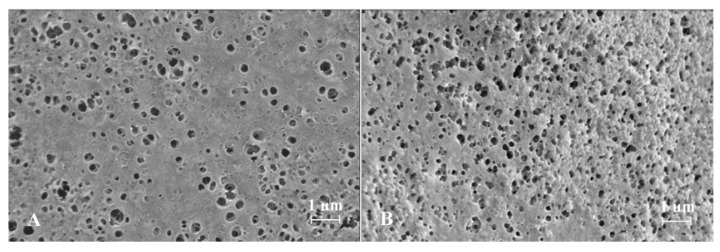
Scanning electron microscopy image of the pores on the surface of spherical in situ forming gels prepared for formulations containing 200 mg eq./g bedaquiline fumarate salt in PLGA5050A/NMP 20/80% (*w*/*w*) (Panel (**A**), formulation 1) and in PLGA7525A/NMP 20/80% (*w*/*w*) (Panel (**B**), formulation 2).

**Figure 3:**
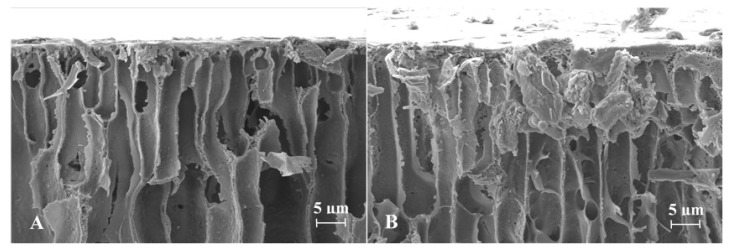
Scanning electron microscopy cross-section image of the finger-like pores at the surface of spherical in situ forming gels prepared for formulations containing 200 mg eq./g bedaquiline fumarate salt in PLGA5050A/NMP 20/80% (*w*/*w*) (Panel (**A**), formulation 1) and in PLGA7525A/NMP 20/80% (*w*/*w*) (Panel (**B**), formulation 2).

**Figure 4:**
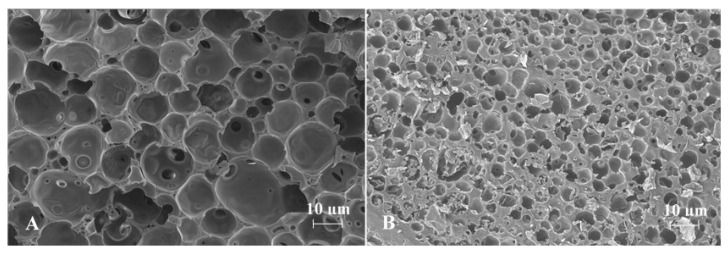
Scanning electron microscopy cross-section image of the sponge-like structure at the inner part of spherical in situ forming gels prepared for formulations containing 200 mg eq./g bedaquiline fumarate salt in PLGA5050A/NMP 20/80% (*w*/*w*) (Panel (**A**), formulation 1) and in PLGA7525A/NMP 20/80% (*w*/*w*) (Panel (**B**), formulation 2).

SEM images obtained for spherical ISGs prepared from formulations 1 and 2 are shown in Figure 2, Figure 3 and Figure 4 (Panel (A) for formulation 1 and Panel (B) for formulation 2).

For both formulations, the spherical ISGs consisted of an outer layer with finger-like pores (Figure 3), of which some formed smaller surface pores (Figure 2), and an inner sponge-like porous structure (Figure 4). The inner pores of the sponge-like structure were larger for formulation 1 compared to those of formulation 2 (Figure 4). A similar trend was observed for surface pores, which were slightly larger and lower in number for formulation 1 (Figure 2).

**Figure 5:**
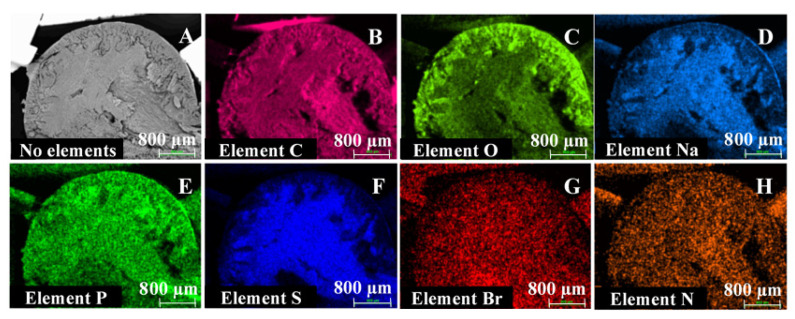
Scanning electron microscopy (SEM) image and energy-dispersive X-ray spectroscopy (EDX) elemental maps of spherical in situ forming gels prepared for formulation 1 containing 200 mg eq./g bedaquiline fumarate salt in PLGA5050A/NMP 20/80% (*w*/*w*). SEM image (Panel (**A**)), EDX maps for carbon coloured pink (Panel (**B**)), oxygen coloured green (Panel (**C**)), sodium coloured blue (Panel (**D**)), phosphor coloured green (Panel (**E**), sulphur coloured blue (Panel (**F**)), bromine coloured red (Panel (**G**)), and nitrogen coloured orange (Panel (**H**)).

**Figure 6:**
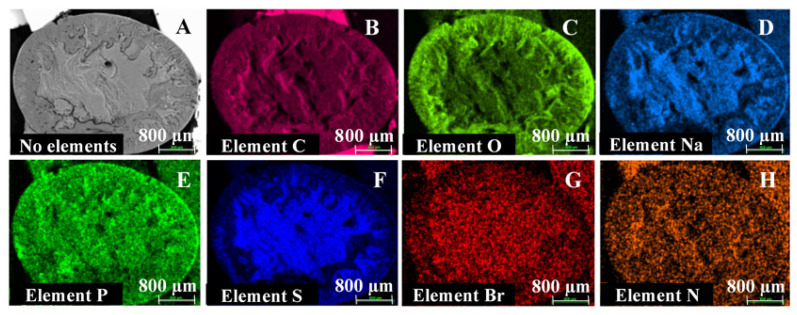
Scanning electron microscopy (SEM) images and energy-dispersive X-ray spectroscopy (EDX) elemental maps of spherical in situ forming gels prepared for formulation 2 containing 200 mg eq./g bedaquiline fumarate salt in PLGA7525A/NMP 20/80% (*w*/*w*). SEM image (Panel (**A**)), EDX maps for carbon coloured pink (Panel (**B**)), oxygen coloured green (Panel (**C**)), sodium coloured blue (Panel (**D**)), phosphor coloured green (Panel (**E**)), sulphur coloured blue (Panel (**F**)), bromine coloured red (Panel (**G**)), and nitrogen coloured orange (Panel (**H**)).

Elemental mappings by SEM-EDX of the cross-section of spherical ISGs formed from formulations 1 and 2 are shown in Figure 5 and Figure 6, respectively.

Carbon (C), present in bedaquiline, PLGA, buffer (SLS), and NMP; phosphor (P), present in buffer (NaH_2_PO_4_×H_2_O); and nitrogen (N), present in bedaquiline and NMP, were homogeneously distributed throughout the spherical ISGs for both formulations. Oxygen (O), mainly present in PLGA and to a lesser extent in buffer (NaH_2_PO_4_·H_2_O, NaOH, H_2_O, and SLS), bedaquiline, and NMP, was highly concentrated in the outer surface of both ISGs. The opposite was observed for the elements sodium (Na) and sulphur (S), present in buffer (NaH_2_PO_4_·H_2_O, NaOH, SLS); and bromine (Br), present in bedaquiline.

### 3.2. In Vivo Pharmacokinetic Study in Rats

#### Pharmacokinetics

**Figure 7:**
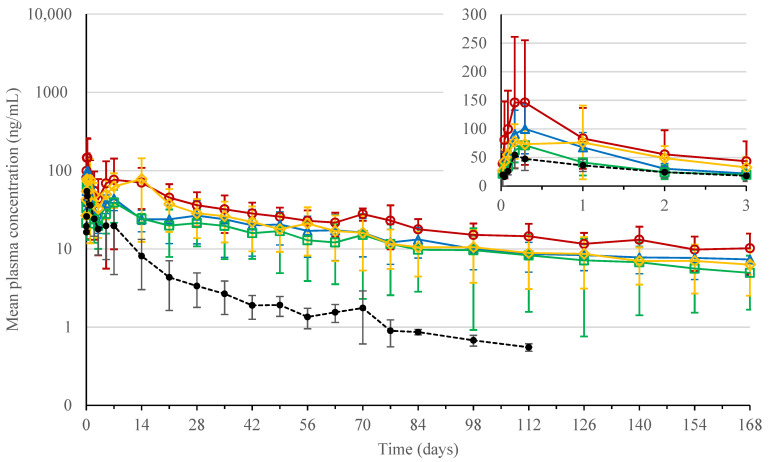
Plasma concentration-time profiles for bedaquiline in rat (mean profiles and standard deviation error bars for *n* = 3). Red circle: in situ forming gel (ISG) formulation 1 (200 mg eq./g bedaquiline fumarate salt in PLGA5050A/NMP 20/80% (*w*/*w*)) dosed subcutaneously (SC) at 80 mg eq./kg; blue triangle: ISG formulation 2 (200 mg eq./g bedaquiline fumarate salt in PLGA7525A/NMP 20/80% (*w*/*w*)) dosed SC at 80 mg eq./kg; green square: ISG formulation 3 (200 mg eq./g bedaquiline fumarate salt in PLGA7525E/NMP 20/80% (*w*/*w*)) dosed SC at 80 mg eq./kg; orange diamond: ISG formulation 4 (200 mg eq./g bedaquiline fumarate salt in PDLLA/NMP 20/80% (*w*/*w*)) dosed SC at 80 mg eq./kg; black dot: solution formulation 5 (5 mg eq./mL bedaquiline fumarate salt in PEG400/water 50/50% (*v*/*v*)) dosed SC at 4 mg eq./kg.

Pharmacokinetic data were obtained for ISG formulations 1 to 4 and PEG400 solution formulation 5 after dorsal SC injection in rats. The administered bedaquiline fumarate salt dose was 80 mg eq./kg for the ISG formulations and 4 mg eq./kg for the PEG400 solution formulation. Figure 7 shows the mean pharmacokinetic profiles, and Table 3 summarizes the pharmacokinetic parameters.

After single SC administration of bedaquiline fumarate salt to male rats, plasma concentrations could still be quantified at the last sampling time point of 4032 h (168 days) post-dose for ISG formulations 1 to 4 (dose of 80 mg eq./kg) and up to 2688 h (112 days) post-dose for the PEG400 solution formulation 5 (dose of 4 mg eq./kg).

Peak concentrations for formulations 1 to 5 were obtained around 4 to 7 h post-dose and less pronounced around 5 to 14 days after dosing.

Formulation 5 showed the fastest initial decline after reaching the second peak concentration, while formulations 1 and 4 showed the slowest decline. From 504 h (21 days) after dosing onwards, the plasma concentration-time profiles of formulations 1 to 4 declined in parallel, indicating a similar terminal t_1/2_ for these formulations (*p* values > 0.05), which was 1.7- to 2.3-fold longer than the terminal t_1/2_ for PEG400 solution formulation 5 (*p* values < 0.05).

Taking into consideration the dose difference, and assuming dose proportional pharmacokinetics as reported for oral dosing of bedaquiline fumarate salt up to 20 mg/kg/day with plasma concentrations up to 1834 ng/mL [35], solution formulation 5 showed a higher C_max_, AUC_72h_ (*p* values < 0.05), and AUC_last_ (*p* values > 0.05) compared to other formulations, indicating a faster absorption or drug release rate. From the ISG formulations, formulation 1 had the highest C_max_, followed by formulations 2, 4, and 3, while AUC_72h_ and AUC_last_ decreased from formulations 1 to 4, 2, and 3, respectively. The differences were not statistically significant (*p* values > 0.05). At 4032 h (168 days) post-dose, the fraction of the bedaquiline dose that reached the systemic circulation ranged from 0.25 to 0.47, compared to PEG400 solution formulation 5.

The intersubject variability for C_max_ and AUC_last_ for solution formulation 5 was 36% and 48%, respectively. The intrasubject variability for ISG formulations 1 to 4 varied from 37 to 71% for C_max_ and from 36 to 55% for AUC_last_.

During the course of the in vivo study, no major clinical observations were reported for the rats in groups 1 to 5.

## 4. Discussion

In vitro release data indicated a significant and variable burst release (8 to 19% within 48 h) with no clear differences between bedaquiline ISG formulations 1 to 4. A relatively high burst release is typical for fast-inverting systems containing water miscible solvents such as NMP, as the solvent diffuses rapidly to the surrounding buffer before formation of a solidified polymer layer at the surface of the gel, thereby releasing a significant amount of dissolved drug [12]. A higher lipophilicity, polymer concentration, and/or molecular weight of the polymer could reduce the burst release [12,15,36]. However, no effect of polymer grade on burst was observed for bedaquiline ISG formulations 1 to 4. Additionally, drug release after burst (>48 h) showed only limited differences for the four ISGs. A slight decreasing trend was observed with increasing hydrophilicity of the polymers (PLGA7525E > PLGA7525A > PLGA5050A) having similar intrinsic viscosity or molecular weight. The opposite could be expected if gel erosion would drive drug release as more lipophilic polymers permit a slower water ingress and thereby slower hydrolysis [12,14]. The dissolution rate observed for ISG formulation 4 with the most lipophilic polymer PDLLA was lower than the one observed for the more hydrophilic polymers PLGA7525E and PLGA7525A, which is likely due to the higher intrinsic viscosity compared to the other polymer grades, reducing the drug diffusion rate [12].

To position the data of this research compared to the available literature, several case studies for ISGs containing lipophilic, poorly water-soluble drugs are discussed. Madhu et al. reported a polymer-erosion controlled drug release of about 85, 75, and 65% within 8 days for 1% (*w*/*w*) rosiglitazone ISGs containing 12.5% (*w*/*w*) PLGA with L/G ratios of 65/35, 75/25, and 85/15, respectively [36]. Additionally, Wang et al. observed release rates of about 75 to 95% within 7 days, in line with polymer hydrolysis rates for 4% (*w*/*w*) risperidone ISGs. However, for 4% (*w*/*w*) paliperidone ISGs, the drug released slower for ISGs with acid versus ester end-capped PLGA, which was attributed to an interaction between the hydroxyl group of paliperidone and the carboxylic moiety of the acid-terminated PLGA, helping drug retention. When the risperidone and paliperidone ISGs were prepared with NMP instead of dimethyl sulfoxide (DMSO), and the drug was primarily dissolved instead of dispersed in the formulations, drug release was much faster and not affected by polymer grade, reaching >70% within 48 h [23]. This highlights the importance of solubilization and/or precipitation behaviour of drugs in the release rate of ISGs. Ahmed et al. described a triphasic in vitro release profile for a 10% (*w*/*v*) haloperidol ISG containing 20% (*w*/*v*) PLGA5050A (IV of 0.5 dL/g) in NMP. A rapid burst phase, releasing 18% haloperidol within the first 24 **h** was followed by a slower diffusion-controlled release phase, lasting for approximately 20 days. Subsequently, a fast erosion-controlled release phase started as soon as the molecular weight of the hydrolysed polymer chains reached a lower threshold [18]. Similar triphasic in vitro release profiles were observed by Xin et al. for 15% (*w*/*w*) hydrochloric thiotixene ISGs containing 32.5 and 45% (*w*/*w*) PDLLA (MW of 55 kDa) in NMP. However, at a lower polymer concentration of 20% (*w*/*w*), only two phases were observed, with a burst release of 30% within 12 h followed by a slower release phase reaching 100% of released drug after 16 days [26].

Drug release of bedaquiline ISG formulations 1 to 4 was biphasic and slower than for the ISGs discussed above. To evaluate if the high drug/polymer ratio (1/0.6) and high drug load (24% (*w*/*w*)) were the reason that polymer erosion did not drive drug release, in vitro release profiles were generated for ISGs with a lower drug/polymer ratio (1/3.9), containing 40 mg eq./g bedaquiline fumarate salt in PLGA5050A or PLGA7525A/NMP 20/80% (*w*/*w*) (data not shown). Likewise, for these ISGs, there was no difference in burst release observed, and the dissolution profile after burst (>48 h) was also slightly slower for the ISG with the more hydrophilic polymer. Another hypothesis for the lack of polymer erosion-controlled release was that rapid precipitation and slow redissolution of the drug predominantly determined the release rate of bedaquiline, independent of the polymer used. Therefore, in vitro release of a solution containing 200 mg eq./g bedaquiline fumarate salt in NMP was evaluated by injecting 0.5 g of the formulation into 900 mL 0.05 M phosphate buffer pH 7.4 with 1% (*w*/*v*) SLS (data not shown). Upon injection, the dissolved drug was distributed throughout the buffer, leading to a burst release of 42% within 15 min after injection. Conversely, part of the drug precipitated instantly into aggregates that slowly redissolved, resulting in a total drug release of only 47% after 11 days. The burst release for ISG formulations 1 to 4 was lower than for the 200 mg eq./g bedaquiline fumarate solution in NMP, as gel formation reduced the drug diffusion rate to the surrounding buffer. After burst, ISG formulations 1 to 4 showed a faster drug release as compared to the solution. This may indicate that the polymers (or their oligo- and/or monomers) act as a surfactant, promoting (re)dissolution of the drug. The solubility of bedaquiline fumarate salt was determined to be higher in 5% (*w*/*v*) lactide versus glycolide in 0.05 M phosphate buffer pH 7.4 (0.019 versus 0.013 mg/mL after stirring for 18 days at 37 °C), suggesting that the surfactant capacity of PLGA increases with increasing lipophilicity, likely explaining the unusual order in release rate for ISGs 1 to 4. Lastly, differential scanning calorimetry (DSC) and infrared spectroscopy (IR) were used to reveal possible interactions between formulation components of the bedaquiline ISGs (data not shown). Theoretically, transesterification between bedaquiline and PLGA could happen. Analysis of spherical ISGs formed from formulations 1 and 2 in 0.05 M phosphate buffer at pH 7.4 or in water indicated the absence of significant molecular interactions. The slower release observed for bedaquiline ISGs compared to ISGs described in the literature is probably due to the high lipophilicity, instant precipitation, and slow redissolution of the drug.

SEM images obtained for spherical ISGs formed in 0.05 M phosphate buffer containing 1% (*w*/*v*) SLS for formulations 1 and 2 (200 mg eq./g bedaquiline fumarate salt in PLGA5050A/NMP or PLGA7525A/NMP 20/80% (*w*/*w*), respectively) visualized an outer layer with finger-like pores surrounding a sponge-like porous structure, as expected for fast-inverting systems containing water miscible solvents, such as NMP [12,37]. The distinctive morphology within these ISGs is the result of a fast solidification of the outer layer caused by an almost immediate solvent exchange at the surface and a slower phase inversion on the inside due to a more progressive penetration of water. Similar structures were reported by Kamali et al. for ISGs containing naltrexone and PLGA in NMP [27] and by Wang et al. for placebo ISGs prepared with PLGA in NMP or DMSO [23]. The surface of bedaquiline ISG formulation 1 showed less pores than that of formulation 2. Pore closure, promoted by a higher polymer chain mobility and lower glass transition temperature (Tg) of the more hydrophilic polymer PLGA5050A in formulation 1, may explain this observation [14,38]. The inner pores were larger for the ISG formed from formulation 1 (diameter ~10 µm) compared to formulation 2 (diameter ~5 µm), which may be attributed to the lower lipophilicity of PLGA5050A, requiring more water ingress before solidification, allowing more time for pore growth, and leading to a faster hydrolysis afterwards [12]. The apparent higher connectivity of the polymer network and larger inner specific surface area for ISG formulation 2 may have contributed to the slightly faster in vitro drug release compared to ISG formulation 1 by increasing the rate of drug solubilization and/or diffusion.

EDX analysis indicated that the outer layer of the ISGs formed from formulations 1 and 2 was polymer-rich based on the observed distribution of oxygen elements, while bedaquiline, visualized by bromine, was not present at the surface of the ISGs. These observations were in line with the typical burst release reported for fast-inverting ISGs. The distribution of the nitrogen elements (present in NMP and bedaquiline fumarate salt) and phosphor elements (present in the SLS containing phosphate buffer used for gel formation) throughout both ISGs suggested a homogeneous ingress of buffer and solvent exchange within the gel matrix. In contrast to phosphor, the sodium and sulphur elements (present in the SLS containing phosphate buffer) were less pronounced in the outer layer of the ISGs, which suggests an interaction between SLS and bedaquiline (e.g., salt metathesis resulting in the formation of bedaquiline lauryl sulphate and sodium fumarate).

In vivo data in rats indicated that ISG formulations 1 to 4 provided an extended drug release compared to the PEG400 solution formulation 5 after dorsal SC injection. However, despite the variation in polymer properties across formulations, all four ISG formulations resulted in a similar in vivo release rate. C_max_ and AUC_last_ were not statistically different between the ISG formulations. It should be noted that based on in vitro data, only minor differences in release rate were to be expected, and given the observed pharmacokinetic variability, sample size was rather low to capture such differences in vivo, if any. As mentioned above, drug substance precipitation, redissolution, and/or diffusion are likely the driving factors for drug release from the bedaquiline ISGs rather than polymer erosion. The fact that the PK profiles resemble those obtained for 200 mg eq./g bedaquiline microsuspensions administered SC to rats supports this thinking [7]. The microsuspensions were prepared with crystalline bedaquiline (free form), while X-ray diffraction (XRD), DSC, and IR analysis of spherical ISGs formed from formulations 1 and 2 in 0.05 M phosphate buffer at pH 7.4 or in water confirmed the presence of amorphous bedaquiline free form and/or salt. For the PEG400 solution formulation 5, partial precipitation of bedaquiline may have occurred as well in vivo, as suggested by the second peak observed in the PK profile. This, together with the long terminal half-life and high tissue distribution reported for bedaquiline after both oral and intravenous administration, should be considered when interpreting the sustained release profiles of ISGs. During the in vivo study, no major clinical findings were reported. As PLGA is known to be a biocompatible polymer, no tolerability issues were expected [15]. However, further toxicological and histopathological evaluations are recommended to determine the safety of the bedaquiline fumarate salt ISGs.

In summary, this case example highlights the importance of the interplay between drug solubility in biological media and dissolution of drug precipitates, which, in addition to the incorporation of diffusion controlling polymers, governs the release of the active drug.

## 5. Conclusions

This research studied in vitro and in vivo drug release of ISGs containing 200 mg eq./g bedaquiline fumarate salt in PLGA5050A/NMP, PLGA7525A/NMP, PLGA7525E/NMP, and PDLLA/NMP 20/80% (*w*/*w*). Despite the variation in lipophilicity of the polymers used, the difference in the in vitro release rate of the four ISG formulations was minimal and not statistically significant in vivo after SC injection in rats. The release rate of bedaquiline ISGs was driven by instant drug precipitation and slow redissolution, rather than polymer erosion. All ISGs resulted in a sustained release in vivo when compared to a solution of bedaquiline fumarate salt in PEG400/water 50/50% (*v*/*v*). In conclusion, PLGA- or PDLLA-based ISGs have shown potential for parenteral sustained delivery of bedaquiline, suggesting further preclinical and clinical studies.

## Figures and Tables

**Table 1:** Formulations prepared for in vitro and/or in vivo assessments.

Formulation Number (Type)	Polymer Grade	Solvent	Bedaquiline Fumarate Salt Concentration	Polymer/Solvent Ratio	Assessments
1 (ISG)	PLGA5050A	NMP	200 mg eq./g	20/80% (*w*/*w*)	In vitro and in vivo drug release, SEM, EDX
2 (ISG)	PLGA7525A	NMP	200 mg eq./g	20/80% (*w*/*w*)	
3 (ISG)	PLGA7525E	NMP	200 mg eq./g	20/80% (*w*/*w*)	In vitro and in vivo drug release
4 (ISG)	PDLLA	NMP	200 mg eq./g	20/80% (*w*/*w*)	
5 (Solution)	PEG400	Water	5 mg eq./mL	50/50% (*v*/*v*)	In vivo drug release

ISG: in situ forming gel; PLGA: poly(lactide-co-glycolide); PDLLA: poly(d,l-lactide); PEG: polyethylene glycol; NMP: *N*-methyl-2-pyrrolidone; SEM: scanning electron microscopy; EDX: energy-dispersive X-ray spectroscopy.

**Table 2:** Pharmacokinetic study design in rats.

Group	N	Formulation Number (Type)	Dosing Route	Dose (mg eq./kg)	Dosing Volume (mL/kg)	Assessments
1	3	1 (ISG)	SC (Day 1)	80	0.4	Pharmacokinetics:0–4032 h (168 days)
2	3	2 (ISG)	SC (Day 1)	80	0.4	
3	3	3 (ISG)	SC (Day 1)	80	0.4	
4	3	4 (ISG)	SC (Day 1)	80	0.4	
5	3	5 (Solution)	SC (Day 1)	4	0.8	

ISG: in situ forming gel; SC: subcutaneous injection.

**Table 3:** Pharmacokinetic parameters in rat following subcutaneous administration of formulations 1 to 5.

Analyte		Bedaquiline				
Species/Sex		Sprague Dawley Rat/Male				
Dosing Route		Subcutaneous				
Formulation Number (Type)		1 (ISG)	2 (ISG)	3 (ISG)	4 (ISG)	5 (Solution)
Dose (mg eq./kg)		80	80	80	80	4
PK parameter:						
n		3	3	3	3	3
C_max_^a^ (ng/mL)	Mean (SD)	152 (109)	100 (44)	71.6 (26.2)	78 (59)	56.5 (20.4)
t_max_^a^ (h)	Min-Max	4.0–7.0	7.0	4.0–7.0	4.0	4.0–7.0
t_last_ (h)	Min-Max	4032	4032	4032	4032	2688
AUC_72h_ (ng·h/mL)	Mean (SD)	5511 (3958)	3639 (1152)	2572 (1199)	4115 (2325)	2186 (807)
AUC_last_ (ng·h/mL)	Mean (SD)	101,258 (44,708)	63,060 (22,519)	52,698 (26,072)	77,457 (42,363)	10,697 (5121)
AUC_∞_^b,c^ (ng·h/mL)	Mean (SD)	123,231 (54,055)	80,651 (26,971)	64,712 (37,540)	88,128 (49,186)	11,344 (4853)
t_1/2_^b,d^ (h)	Mean (SD)	1695 (275)	1980 (594)	1606 (210)	1411 (122)	854 (264)
C_max_/Dose (10^−6^/mL)	Mean (SD)	1.90(1.36)	1.25 (0.54)	0.895 (0.328)	0.974 (0.379)	14.1 (5.1)
AUC_72h_/Dose (10^−6^·h/mL)	Mean (SD)	68.9 (49.5)	45.5 (14.4)	32.1 (15.0)	51.4 (29.1)	547 (202)
AUC_last_/Dose (10^−6^·h/mL)	Mean (SD)	1266 (559)	788 (281)	659 (326)	968 (530)	2674 (1280)
AUC_∞_/Dose (10^−6^·h/mL)	Mean (SD)	1540 (676)	1008 (337)	809 (469)	1102 (615)	2836 (1213)
AUC_last_/Dose vs. F5	Ratio of means	0.47	0.29	0.25	0.36	-
C_max_/Dose vs. F5	Ratio of means	0.13	0.09	0.06	0.07	-

ISG: in situ forming gel; SD: standard deviation; Min: minimum; Max: maximum. ^a^ C_max_ and t_max_ corresponding to the first peak in plasma concentration-time profile; ^b^ R^2^adj ranged from 0.73 to 0.98 for in situ forming gel (ISG) formulations 1 to 4 and from 0.66 to 0.90 for polyethylene glycol 400 (PEG400) solution formulation 5; ^c^ extrapolation for AUC_∞_ ranged from 10 to 30% for ISG formulations 1 to 4 and from 2 to 11% for PEG400 solution formulation 5; ^d^ t_1/2_ was calculated from 504 h (21 days) after dosing up to t_last_.

## Data Availability

The data presented in this study are available on request from the corresponding author.
